# Ready for Online? Exploring EFL Teachers’ ICT Acceptance and ICT Literacy During COVID-19 in Mainland China

**DOI:** 10.1177/07356331211028934

**Published:** 2022-03

**Authors:** Bingqing Li

**Affiliations:** 1 Faculty of Education, Monash University, Melbourne, Australia

**Keywords:** emergency remote teaching, ICT integration, ICT literacy, ICT acceptance, EFL teachers, COVID-19 pandemic

## Abstract

This study sought to understand whether and how a group of English as a foreign language (EFL) teachers in mainland China were ready for the emergency remote teaching triggered by the COVID-19 pandemic and explains the underlying mechanisms that influenced their readiness. The urgency of the sudden shift to online teaching during the COVID-19 pandemic necessitated teachers' acceptance and literacy of technologies, but so far, little study has touched on this area. Based on the Technology Acceptance Model and the Technological Pedagogical and Content Knowledge conceptual frameworks, a mixed-methods online survey was designed to collect data among 186 high school EFL teachers in China about four months after the governmental response plan of online teaching. The results show participants were overall affirmative in acceptance and knowledge, implying their general readiness for the ICT use in COVID-19 emergency remote teaching. However, there were pressing problems with integrating technology with pedagogy and subject teaching. This study extends previous research by helping understand teachers' most recent adoption of ICT during an unprecedented crisis, informing future ICT training, and predicting their future technological behaviour.

The new coronavirus pandemic, officially named COVID-19, has posed unprecedented challenges to education worldwide. Since its outbreak in December 2019, approximately 1.7 billion students in over 190 countries (almost 90% of total enrolled learners) had been displaced (UNESCO, 2020) to contain the spread of the disease. Massive school closures had interrupted students' learning so harshly that emergency response plans had to be explored to ensure the continuity of education worldwide (Adedoyin & Soykan, 2020).

Information and communication technology (ICT) has been playing a predominant role in responding to the interruption of education during COVID-19 (Bhaumik & Priyadarshini, 2020; Chakma et al., 2021). It provided a safe, timely, and flexible way for educational institutions to sustain school operations (Gacs et al., 2020). Via ICT such as online platforms, social media, and broadcasts, emergency remote teaching (ERT) was quickly offered, transferring traditional face-to-face teaching to virtual spaces. It seemed that ICT integration was no more a choice for quality teaching but a necessity of education in this pandemic. However, doubts soon arose about whether teachers were ready for the sudden switch to the ERT during the COVID-19 pandemic (COVID-19 ERT; Talidong, 2020), which inevitably required teachers' higher digital competence in terms of ICT knowledge, skills, and attitudes (Adedoyin & Soykan, 2020). This study attempts to problematise such doubts and critically examine how some of these can be mitigated in the context of China, the first country to take extreme measures to close all schools. Specifically, this research looks at a cohort of English as a foreign language (EFL) teachers in high school to understand their perceptions and knowledge of ICT during COVID-19.

In the following sections, after a review of literature on ERT and teachers' readiness, research gaps are identified, followed by the proposed research questions. By adopting the mixed-methods approach, the findings from a survey are then presented and discussed. Finally, the article concludes with suggestions for teacher educators to consider teachers' demands in integrating subject matters and pragmatics with ICT in a more efficient manner.

## Literature Review

This section first presents the nature of ERT and the potential challenges for teachers to transit from traditional ICT blended courses to ERT, especially during COVID-19. It, therefore, highlights the significance of focusing on teachers' ICT readiness, which is still an underexplored area. Following this, the concept of ICT readiness is disentangled concerning two interrelated aspects: ICT acceptance and ICT literacy.

### Emergency Remote Teaching

The recent outbreak of the COVID-19 pandemic has led to a renewed interest in ERT, a temporary crisis-prompted educational response with the primary objective of continuing normal teaching online (Chowdhury & Sarkar, 2018; Hodges et al., 2020). Generally, ERT involves a wide use of technologies (Hodges et al., 2020), such as synchronous communication tools (e.g., course delivery platforms and social media), asynchronous platforms for collaboration, and online electronic resources (see Table 1; Hodges et al., 2020). Various ICTs collaborate to build a virtually intact school and to fulfil regular face-to-face teaching and learning activities.

**Table 1.** Major Tools for COVID-19 ERT in China.

Types	Chinese platforms
Course delivery tools	DingTalk, Tencent Classroom, Zoom
Social media	WeChat, QQ
Online learning environments	Moodle, SmartLearning
Evaluation	Yuantiku
E-resources	Eduyun
Collaboration tools	Tencent Document

Transitioning to ERT can hardly be easy for many teachers, especially within the short time for planning, preparedness, and development of ERT (Gacs et al., 2020; Hodges et al., 2020). During COVID-19, for instance, teachers rushed to deliver online courses a few weeks after school closure, in sharp contrast with the suggested months or a year of planning for online courses (Owusu-Fordjour et al., 2020). Meanwhile, teachers have to make a significant leap in ICT integration (Gacs et al., 2020) to bridge the gaps between regular blended courses to ERT. For example, teachers might need new methods to provide timely feedback, monitor students' online learning, or provide personalised study/learning options (Zhou et al., 2020), because a previously fixed study timetable was possibly broken by online discussion forums and recorded classes, in which students can participate and review anytime they prefer (Hodges et al., 2020). In this sense, a smooth transition to ERT needs teachers to explore the usefulness of various ICT, acquiring new knowledge to be familiar with online platforms, and developing novel strategies for integrating ICT in teaching. That is, ERT inevitably necessitated teachers' readiness for ICT (Bhaumik & Priyadarshini, 2020).

Ideally, teachers should have well-informed acceptance and be prepared with Technological Pedagogical and Content Knowledge (TPACK) knowledge before they take the role of online instructors. However, more frequently, the development of teachers' readiness for ERT is an *ongoing* and *adaptive* process because the time for preparation is insufficient, and teachers need to address new problems that may expose in practice. This dynamic nature of ICT integration in ERT reinforces the importance of evaluating teachers' readiness and offering subsequent continuing professional development to improve the effectiveness of ERT. It is particularly true for China during COVID-19, considering its rapid transition nationwide, huge population and unbalanced ICT development (Huang et al., 2019).

However, since the COVID-19 ERT scenario is still a current issue, only a few studies into the teachers' readiness have so far been published, even fewer so in the Chinese context. For example, Talidong (2020) conducted an online survey to examine the readiness for COVID-19 ERT among 20 Philippine teachers who taught EFL in Chinese primary schools. This empirical research is a good attempt to investigate the implementation of ERT in the Chinese EFL context and suggests that in China, foreign EFL teachers may develop a high level of acceptance and TPACK perceptions for ERT. Nevertheless, there is still little research on Chinese local EFL teachers' ICT readiness, and even less is theoretically based. This research aims to address the gaps, and the next section discusses two theoretical frameworks of ICT readiness.

### Teachers’ ICT Readiness: ICT Acceptance and ICT Literacy

ERT needs teachers' *readiness* for ICT because it could strongly influence the speed and quality of ICT integration (Petko et al., 2018; Tondeur et al., 2017). Here, by following the definition proposed by Petko et al. (2018), the term ‘readiness' generally covers two aspects: teachers' acceptance of ICT and their ICT literacy. So far, different models and theoretical frameworks have been proposed to measure readiness concerning these two aspects. Among them, the commonly used and well-accepted frameworks are the Technology Acceptance Model for ICT acceptance and Technological Pedagogical and Content Knowledge for ICT literacy, which are applied in this study and explained below.

### ICT Acceptance: Technology Acceptance Model

**Figure 1.**
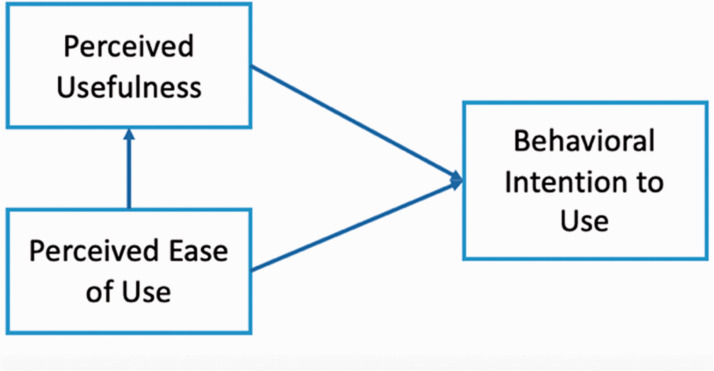
A Diagram for TAM.

The first aspect of readiness is technology acceptance, referring to “a user's willingness to employ technology for the tasks it is designed to support” (Teo, 2011, p. 1). It takes psychological constituents as major predictors of teachers' technology adoption, assuming that their intentions influence their actual use of technology. Technology Acceptance Model (TAM), proposed by Davis et al. (1989), is a dominant and well-established model to explain the mechanisms of teachers' acceptance of technology (Scherer et al., 2019). It clarifies the causal relationships of internal factors, as shown in Figure 1.

In TAM, two main variables predict an individual's technological behaviour: “Perceived Usefulness” (PU) and “Perceived Ease of Use” (PEU). PU is defined as the degree to which the technology enhances the performance of users (Davis et al., 1989), such as better student-teacher interaction, while PEU refers to the amount of effort that a person considers making when using a particular technology (Davis et al., 1989). These two variables directly influence teachers' intention of technology use. Although attitude was regarded as an intermediate variable between PU, PEU, and intention of use in the first version of TAM, Davis and Venkatesh (1996) excluded it from the model since the causal relationship was not confirmed in empirical research. Therefore, in this study, only PU and PEU are included as predictors of teachers' acceptance.

Data from several studies suggest that Chinese EFL teachers were generally affirmative in PU and PEU (e.g., Hsu, 2016; Teo et al., 2018). They were prone to perceive the advantages of technology, such as better online interactions (Hu & Mcgrath, 2011). However, such positiveness was not prevalent in all studies. Evidence shows that some Chinese EFL teachers did not always use technology as predicted (H. Liu et al., 2019), and despite the initial optimistic expectation, their enthusiasm in ICT decreased because of a lack of skills, support, and training (Hu & Mcgrath, 2011).

### ICT Literacy: Technological Pedagogical and Content Knowledge

Another aspect for ICT readiness is ICT literacy, a “measure of an individual's ability to use digital technology”, which focuses on a set of skills using a range of technologies (Mac Callum et al., 2014, p. 9). TPACK model echoes this concept and has become more frequently adopted in the research on ICT literacy (see, e.g., Tondeur et al., 2020; Yanuarto et al., 2021). Besides, while TAM is a technocentric model for ICT acceptance, TPACK supplements the understanding of teachers' ICT use with more pedagogical concerns of teachers' ICT knowledge (Harris et al., 2009), which concords with the subject-specific standpoint of the research questions. For these reasons, TPACK is chosen as a suitable guideline for this research.

**Figure 2.**
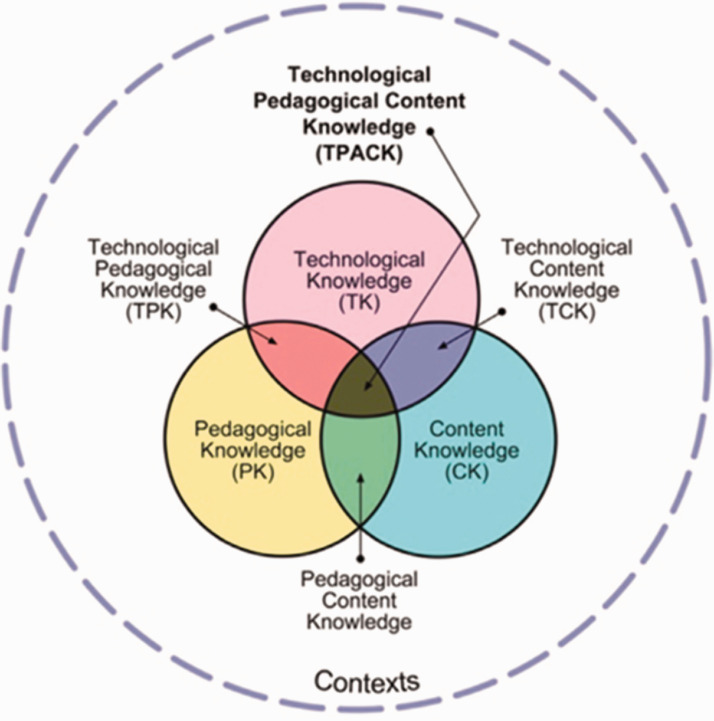
A Diagram for TPACK (Adapted From Mishra & Koehler, 2006).

Developed from the notion of Pedagogical Content Knowledge (PCK) proposed by Shulman (1986), which explains the interplay of pedagogy knowledge (PK) and content knowledge (CK), Mishra and Koehler (2006) added technology knowledge (TK) as the third core constituents to explain the nuanced cognitive procedure of teaching. The interaction of these three primary components gives rise to secondary forms of knowledge: pedagogical content knowledge (PCK), technological content knowledge (TCK), technological content knowledge (TPK), and ultimately the synthesised constitute—TPACK (Mishra & Koehler, 2006), see Figure 2. In this model, knowledge of both technology and educational factors (PK and CK) interweaves to provide effective teaching with ICT. To explore teachers' knowledge of technology, four variables in the TPACK framework (TK, TPK, TCK, and TPACK) are selected for this research.

Despite the recent increase in research of TPACK, only a few studies focused on the examination of TPACK among Chinese teachers, even less on EFL. The existing literature reported that Chinese EFL teachers had overall positive perceptions of TPACK (Chai et al., 2013; Q. Liu et al., 2015 ). However, X. Liu et al. (2014) cautioned that their TPACK perceptions remained unsatisfactory to support the instruction in language teaching. Q. Liu et al. (2015) further confirmed this result, indicating that some K-12 EFL teachers in China found it hard to integrate technology in their teaching and content representation.

### Connections Between ICT Acceptance and ICT Literacy

Given the common target of ICT acceptance and ICT literacy to prepare teachers for effective ICT integration, recent researchers have further drawn on the associations between these two dimensions through the lenses of TAM and TPACK to increase the understanding of the underlying mechanism of ICT integration. Up to now, several studies have agreed that the synthesised component TPACK could be a predictor for ICT acceptance. For example, Hsu (2016) found that TPACK positively affected PEU and PU in technology by partial least square analysis among Taiwanese secondary EFL teachers. Using an analysis of structural equation modelling, Yang et al. (2019) supported these findings in their study of Chinese in-service K-12 teachers. This preliminary research has agreed that the component TPACK could be a predictor for acceptance of ICT, enhancing the understanding of the theories in acceptance and TPACK.

Research on the connection between TAM and TPACK has grown in importance due to the outbreak of COVID-19. As discussed earlier, effective COVID-19 ERT entailed teachers' rapid development in both ICT acceptance and ICT literacy. If taking TAM and TPACK models into consideration, it means teachers were supposed to increase their PU, PEU, TK, TCK, TPK and TPACK. A clear understanding of the interrelations among these psychological and cognitive factors may inform an efficient way of ICT training and support. However, previous expositions may be unsatisfactory because much uncertainty still exists about the reciprocal relationships between ICT acceptance and other subscales in the TPACK framework (e.g., TK, TPK, and TCK). Also, there is little published data on high school EFL teachers in mainland China. Therefore, more variables need to be discussed to explain further the complicated mechanism of ICT use among EFL teachers in mainland China, which will be explored in this research.

## Purpose of the Study

So far, two research gaps are identified in the literature: 1) there is a lack of research on Chinese EFL teachers' readiness (ICT acceptance and ICT literacy) for ERT, particularly during the COVID-19 pandemic; 2) there is existing uncertainty of relationships between TPACK components (especially TK, TPK, and TCK) and TAM. This research aims to fill these gaps by conducting an online survey to explore *how teachers responded to the ICT integration in COVID-19 ERT* in the Chinese EFL setting. Specifically, this study answers three research questions:

1. What is high-school EFL teachers' acceptance of ICT in Mainland China during COVID-19 ERT?
2. What is high-school EFL teachers' ICT literacy in Mainland China during COVID-19 ERT?
3. What are the correlations between their ICT acceptance and their ICT literacy?

This research study is of significance for several reasons. Of prime importance, this empirical study helps understand EFL teachers' ICT readiness in terms of acceptance and literacy in an unprecedented crisis of COVID-19. It should be noted that ICT readiness is not fixed or innate; instead, it is a relative, fluid and developmental construct, which may be influenced by the change in the context. Therefore, although there have been extensive examinations of the impact of beliefs and knowledge on ICT behaviour, it seems these dimensions may be re-shaped by the urgency of the pandemic, the intensive training before and during COVID-19 ERT, the growing demands for ICT and teachers' more frequent usage of ICT in ERT compared with traditional blended courses (Gacs et al., 2020). For these reasons, I believe it is worth investigating the updated condition of teachers' ICT integration in ERT.

In addition, this study provides important implications on how to prepare Chinese EFL teachers in high schools for integrating ICT in the future, which remains uncertain due to the amorphous nature of the ongoing pandemic. Answering the first two research questions can inform in which aspects teacher educators should give extra support in the following training. Besides, an investigation to the third research question will advise future ICT training more effective approaches to nurturing teachers ICT readiness in a way that professional development programs may teach subtypes of TPACK knowledge alongside an explanation of PU or PEU. Further, because readiness could predict their future technology use (Inan & Lowther, 2010), this study of Chinese EFL teachers' readiness could suggest the influence of COVID-19 ERT on teachers' future technological behaviour. Is it a valuable experience promoting future integration of online teaching in the long run (Zhou et al., 2020)? Or should more efforts be made to avoid the misconception of online learning as an ineffective teaching mode? Teachers' voices will generate fresh insights to understand how COVID-19 ERT will influence future integration.

## Methodology

### Research Design

**Figure 3.**
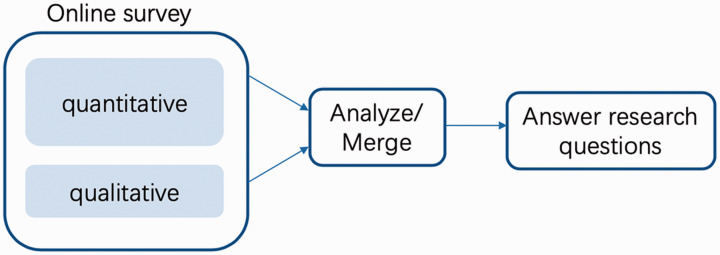
Mixed Methods Design.

This research adopted a convergent QUAN+qual mixed methods design (Creswell & Creswell, 2018), as shown in Figure 3, with the quantitative method as the principal data-gathering tool. By following this design, both quantitative and qualitative data were collected concurrently in a single phase. Due to this study's descriptive and theory-testing nature and purposefully a large sample, the quantitative method was the principal data-gathering tool. The quantitative and the qualitative databases allow to be cross-checked and compared (Bryman, 2016), offering more comprehensive and valid answers to the research questions (Creswell & Creswell, 2018).

### Instrument Design

The online survey included four parts (29 items) for collecting quantitative and qualitative data. Part 1 elicited participants' demographic information, including background information (gender, age range, grade(s), teaching experience), basic ICT use, and professional development during the COVID-19 pandemic.

The second part of the survey collected information about respondents' PU and PEU to answer the first research question based on TAM. The items were adopted from the instrument developed by Teo (2011) because it is specific to EFL language teachers and has been validated in the Chinese context by H. Liu et al. (2018).

The third part examined the four constitutes of TPACK (TK, TCK, TPK, and TPACK) for the second research question. Items were selected from three instruments related to EFL TPACK, designed by Baser et al. (2016), Bostancıoğlu and Handley (2018), and Schmidt et al. (2009). Three instruments are tested to be reliable (Cronbach's α > 0.80). However, some items in these instruments were excluded in this survey since they focus on preservice teachers in other subjects instead of EFL in service teachers. Some other items were modified to suit Chinese EFL teachers. For instance, following the ICT tools used by Chinese teachers, some items of TK were changed, such as “I know how to use computer-mediated communication technologies (e.g. email, chat)” into “I know how to use interactive communication technologies (e.g. WeChat and QQ)”.

In contrast to the fixed-choice questions in Parts 2 and 3, the fourth part asked EFL teachers an open-ended question (”If I have one wish for using technology for teaching EFL, it is…”). This question helped to understand EFL teachers' present situation in COVID-19 ERT, suggesting what they desired for pedagogical technology use but perhaps had not attained yet (Wilkie, 2019), their concerns of technology use or suggestions for improvement might be elicited. In addition, it gave participants a greater opportunity to express themselves spontaneously in the time of transformation to online teaching as a supplement to the closed-ended questions in Parts 2 and 3. Methodologically, this question makes the study a mixed-methods one since responses to this open-ended question were all unique by nature.

### Data Collection and Participants

After this project obtained Ethics Approval, the online survey was made available on Qualtrics on 10th May 2020. By following snowball sampling (Bryman, 2016), the initial participants (from the researcher's personal contact, the EFL teachers who participated in a curriculum training program and the alumni of a teacher training university) were invited through WeChat, a Chinese social platform. Once consent was attained for the initial participants, they were encouraged to invite anyone eligible to the survey.

A total of 195 Chinese high school EFL teachers finally participated in the online survey and provided 186 valid responses. Nine cases were removed for missing values after performing Missing Value Analysis and Little's MCAR test (χ^2^ = 111.563, *df* = 137, *p* = 0.946), which inferred that the data were missing completely at random.

### Data Analysis

For quantitative data, items were designed for these factors. The five-point Likert scale used in P2 and P3 was coded as 1 = *strongly disagree*, 2 = *somewhat disagree*, 3 = *neither agree nor disagree*, 4 = *somewhat agree*, 5 = *strongly agree*. Then, after the pre-analysis of the data, descriptive statistics (mean and standard deviation, percentage) were obtained from the software package SPSS 24.0. Tabulations helped to understand the characteristics. Finally, bivariate correlations were computed by Pearson's correlation analysis.

The qualitative data was analysed via directed content analysis (Hsieh & Shannon, 2005), in which the initial codes were six pre-determined variables or themes. In this case, after reading the data twice, the data was coded by bracketing chunks and writing key words in each comment. The reoccurred key words were regarded as sub-themes, and then grouped into six themes. The analysis was checked twice, after the first-round coding and after coding all the answers.

Then, the qualitative data and quantitative data were analysed jointly, following a side-by-side approach of data integration (Creswell & Creswell, 2018) to produce an analytical density and triangulation. Specifically, qualitative data and quantitative data were compared, contrasted, and/or synthesised (Creswell & Creswell, 2018) concerning the variables of ICT acceptance and ICT literacy (PU, PEU, TK, TCK, TPK, TPACK).

The researcher has adopted four strategies to enhance the trustworthiness of the qualitative analysis (Lincoln & Guba, 1985). First, the researcher had developed early familiarity with the culture of the EFL teachers' adoption of ICT before the researcher started to collect data. Also, a colleague has been invited to scrutinise the research project and to comment on the process. In addition, frequent de-briefing sessions were organised with an expert mixed-methods researcher. Finally, the researchers' reflective commentary was kept to record the process of choice-making, which provide possibilities for monitoring and reflecting the researcher's interpretation of the data and assumptions.

## Findings

### Demographic Information

There were 125 female participants (67.2%) and 61 male participants (32.8%). The majority of participants were below 40 years old (*n*=134, 72%). Consistently, over half of the participants (*n* = 99, 53.1%) had less than ten years of teaching experience. In terms of year level, 116 EFL teachers (62.4%) taught in junior high school (from Year 7 to Year 9 in China); five taught multiple grade levels. Meanwhile, 70 EFL teachers (37.6%) were senior high school teachers (from Year 10 to Year 12), with five teaching multiple grade levels. Compared with national statistics (Ministry of Education [MoE], 2020b, 2020c), the composition of participants is similar to that of the population in terms of gender, grade levels, but there were more participants less than 40 years old and with less than ten years of teaching experience.

Participants named 34 online platforms that they used during COVID-19 ERT. Among them, the leading type was communication platforms such as DingTalk (*n* = 144, 77.4%) and Tencent classroom/meeting (*n* = 52, 28%). Besides, social media played an important role, with over half reporting using WeChat (51.1%, *n* = 95) and 28% of teachers (*n* = 52) using QQ. Also, 54 teachers (29%) used Eduyun, a free national online cloud classroom launched by MoE after the COVID-19 outbreak (MoE, 2020a). In terms of how participants learnt to use these online platforms during COVID-19, all participants had at least one approach of ICT training. Formal training workshops from schools were the most common way, with 139 EFL teachers (74.7%) choosing it. Online resources and informal collaboration with colleagues also contributed to the familiarity with the tools for nearly half of the participants (*n* = 92, 49.5%; *n* = 90, 48.4%).

### Quantitative Data Analysis

#### Validity and Reliability

Items for TPACK and TAM constituents were subjected to an Exploratory Factor Analysis (EFA) with Oblimin rotation to evaluate the validity. After examining the suitability of data, an inspection of the scree plot and parallel analysis yielded a two-factor solution for TAM and a three-factor solution for TPACK as the best fit for the data. The two factors for TAM suited the original variables in the TAM survey: PEU and PU, with factor loading range between 0.63 and 0.90. As for TPACK, four items of TK and four of TCK had the same structure as in the original scale. Factor loading ranged from 0.55 to 0.81. However, the original items for TPK and TPACK combined as one, suggesting that the EFL teachers were unable to distinguish the items in these two groups. The new factor was renamed TPK-TPACK in the following analysis. The instruments of TPACK and teacher's acceptance had good internal consistency as the values of Cronbach alpha for TAM and TPACK were acceptable, at 0.87 and 0.77 (Pallant, 2016), and those values for factors extracted from EFA were all above 0.70.

#### EFL Teachers’ Acceptance and TPACK Perceptions

The mean scores for PU and PEU were 3.50 and 4.05, respectively, with standard deviations of 0.82 and 0.64, both significantly higher than the average 3.00 via one-sample *t*-test, suggesting that ICT use gained general acceptance among the EFL teachers during COVID-19 ERT. A paired sample *t*-test demonstrated there was a statistically significant difference between PU and PEU, *t* (185)=8.830, *p* < 0.001 (two-tailed). The mean difference was 0.55, with a 95% confidence interval ranging from 0.44 to 0,67. It means that the participants perceived more easiness than usefulness when using ICT during the COVID-19 pandemic.

The mean scores of three factors of TPACK constituents were above 4.00, revealing overall positive perceptions of TPACK. The participants perceived themselves strongest in TK, with the average score reaching 4.19, but relatively equally weak in TCK, TPK-TPACK, 4.02 and 4.01, respectively. A series of paired *t*-tests showed the significant difference between TK and TCK, *t* (185)=4.14, *p* < 0.001 (two-tailed), the mean difference was 0.19 with a 95% confidence interval from 0.10 to 0.28; and between TK and TPK-TPACK, *t* (185)=4.34, *p* < 0.001 (two-tailed), the mean difference was 0.17 with a 95% confidence interval from 0.09 to 0.24.

#### Correlations Between TPACK and Acceptance of ICT

The results of the bivariate analysis of TPACK and acceptance suggest that positive correlations between every two variables were established at *p* < 0.01, as shown in Table 2. The correlation coefficients varied between *r* = 0. 32 and *r* = 0.61, within the range of medium and large (Pallant, 2016), revealing general correlations between acceptance and TPACK perceptions. There were two strong positive correlations (*r* ≥ 0.5; Pallant, 2016): one was between TK and PEU (*r* = 0.61), and the other was between TPK-TPACK and PU (*r* = 0.50).

**Table 2.** Pearson Product-Moment Correlations Between Measures of Acceptance and TPACK.

	PU	PEU
TK	0.32**	0.61**
TCK	0.39**	0.42**
TPK-TPACK	0.50**	0.45**

*Note*. Correlation coefficients above 0.50 are in bold.

**Correlation is significant at the 0.01 level (2-tailed).

### Qualitative Data Analysis

Out of 186 responses, 137 participants answered the open-ended question in Part 4. As summarised in Table 3, the results show that two dimensions (acceptance and TPACK, including six pre-determined constructs) all emerged in EFL teachers' answers. More comments were about TPACK (*n* = 77, 56.2%) than acceptance (*n* = 46; 33.6%), indicating that teachers might expect to learn how to use ICT than accepting it. It should be noted here that although the primary intention for this one wish question was to elicit expectations for future improvement in ICT integration, most comments within the TPACK dimension are *concerns* (dissatisfaction with current situations), except for the TK category, as discussed below.

**Table 3.** Themes and Sub-Themes From the Open-Ended Question.

Dimension	Theme	*n*	Sub-theme (*n*)
TPACK	TPK	50	Interaction (18);
			Monitor (11)
			Feedback (10)
			Teaching resources (8)
	TPACK	11	General (4)
			Specific English skill (7)
	TCK	9	Speaking (4)
			Environment (3)
			Translation (2)
	TK	7	Familiarity (5)
			Specific ICT (2)
Acceptance	PU	26	Efficiency/ Quality (15)
			Specific affordance (6)
			Negative (5)
	PEU	20	Easy to apply (13)
			Easy to learn (7)

The most constant theme for the TPACK dimension was the demand for higher TPK (*n* = 50, 36.4%). Within this category, concerns for ICT use in classroom management, especially for online interaction, were more widespread. Eighteen participants (13.1%) argued that ICT should enable more synchronous, teacher-student, and student-student interaction in ERT. One comment was “I hope teachers can direct talk to students, like in real classes”. It seems that computer-mediated communication did not satisfy these EFL teachers' needs to interact with students. The teachers expected ICT could enable similar interaction with face-to-face teaching so that, possibly, their pedagogical strategies could be applied. In addition, feedback on assignments and teaching materials were the other two sub-themes of TPK. Some participants hoped that ICT could help enhance the effectiveness of giving feedback and the quality of textbooks.

Other themes of TPACK also emerged, with 11 teachers expressing their concerns for TPACK (8.0%) and nine for TCK (6.5%). These comments involve teachers' expectation of applying technologies in subject teaching, mainly to improve speaking, language environment and translation in language teaching. Lastly, there were seven wishes (i.e., positive expectations) relevant to TK (5%), among which five teachers wanted to their familiarity with ICT, while two teachers wanted to use PowerPoint or blackboard in online courses.

For the second dimension - teachers' acceptance - a total of 26 participants (18.9%) described their wishes about PU, while 22 participants (16.0%) about PEU. The responses in PU mainly involved teachers' wishes for more efficient teaching with ICT and their perceived affordance for particular ICT, such as artificial intelligence and online forums. Meanwhile, the participants described their wishes for PEU related to ‘easy to learn' and ‘easy for use', such as “more convenient”, “easier”, or “more efficient”, “greater integration”. Comparative adjectives reoccurred in these responses of PEU, which, to some extent, indicate that the EFL teachers admitted the low threshold of ICT but hoped for much easier applications for them to learn and apply so that the integration of ICT could promote the quality and effectiveness of their teaching.

Besides, notable was that, different from all the positive expectations for future development in PEU, some participants voiced their doubts about the effectiveness of ICT (i.e., PU of ICT in ERT). For example, one EFL teacher responded: “ICT can only be a supplementary tool for teaching, and its use should be more than a mere formality”. “Formality” may suggest that some EFL teachers were not motivated in ICT integration but had passively adopted ERT due to official requirements or a compulsory educational response to the crisis. They did not fully support the ICT use in ERT and might consider that the importance of ICT was exaggerated during ERT. These comments also indicate that somehow, teachers admitted the effectiveness of ICT as a “supplementary tool” instead of the only way of course delivery, implying that their PU of ICT could be different between blended courses and ERT.

## Discussion

### RQ1: High-School EFL Teachers’ Acceptance of ICT

This study set out with the first research question of assessing the teachers' acceptance of ICT, which was discussed in P2 and P4 of the survey. By synthesising quantitative and qualitative results, as shown in Table 4, it was found that ICT was generally viewed as useful and easy to use during COVID-19 ERT – considering the overall higher score in PEU and more frequent positive comments in learning to use ICT. These results align with previous findings in the literature that Chinese language teachers had positive perceptions of PU and PEU (see, e.g., Hsu, 2016; Huang et al., 2019; H. Liu et al., 2018 ).

**Table 4.** Integrated Results Matrix for Teachers’ Acceptance.

	Quantitative results	Qualitative results
PU	Mean = 3.5	n = 26; most positive comments
PEU	Mean = 4.05	n = 20; overall positive comments

One marked result from the quantitative data is that the EFL teachers, on average, rated PEU higher than PU in this research. This is in contradiction with previous results reported in the literature. For example, Mei et al. (2018) reported that the average scores for PU were significantly higher than those for PEU. One possible explanation for this discrepancy could be that Mei and his colleagues focused on preservice EFL teachers in China while this study in-service high school EFL teachers. As Hsu (2016) points out, different subgroups of EFL teachers may have diverse perceptions of ICT.

In addition to the differences among groups of EFL teachers, another reason for the change in sub-categories of ICT acceptance might be that EFL teachers have different perceptions of blended courses and ERT. In previous research, EFL-teacher participants primarily used ICT as a supplementary tool for their face-to-face teaching, whereas in the context of COVID-19 ERT, all the teaching and learning activities are fulfilled simply via ICT (Hodges et al., 2020). The sudden change to this entirely ICT-based teaching environment may result in the problem that some traditional face-to-face teaching strategies might not be effectively adopted in this new teaching mode. When this happens, it is possible to assume that teachers are likely to blame it on ICT, believing ICT fails to enable them to deliver courses fluently. This so-called “stakeholder resistance”, as explained by Gacs et al. (2020, p. 3), possibly leads to a decrease in teachers' perceived usefulness for ICT. As indicated in the responses of the “one wish” question, not “*like in real class*” was teachers' explanations for their dissatisfaction with using ICT during interaction with students. In other words, teachers harboured specific concerns and misgivings for the usefulness of online teaching in fully online teaching mode due to the challenges of new teaching strategies in ERT. Therefore, in order to promote EFL teachers' acceptance of the affordances of ICT, a helpful recommendation to consider could be to help teachers develop innovative teaching strategies with ERT.

### RQ2: High-School EFL Teachers’ ICT Literacy

The second question in this study sought to assess EFL teachers' TPACK perceptions. As shown in Table 5, quantitative results of P2 disclose that the EFL teachers had overall positive perceptions of TPACK (means over 4.0), which may imply that professional development trained EFL teachers with general TPACK knowledge that they needed to implement ERT. This result corroborates the findings of previous research on Chinese teachers' overall positive TPACK perceptions (e.g., Hsu, 2016; Q. Liu et al., 2015), although these previous studies were not conducted during a pandemic scenario.

**Table 5.** Integrated Results Matrix for TPACK Perceptions.

	Quantitative results	Qualitative results (n; sub-themes)
TK	Mean = 4.19	n = 7; Familiarity
TCK	Mean = 4.01	n = 9; Speaking/ translation/ environment
TPK-TPACK	Mean = 4.02	n = 50; TPK: Classroom management/ feedback/ resourcesn = 11; TPACK: specific English skills

Comparing the components of TPACK, it is interesting to note that the EFL teachers aligned themselves more positively with TK and relatively weakly with TCK, TPK-TPACK. In the quantitative results, TK scored highest while the other three scored lower. Teachers' comments also reflect that the participants' difficulties of ICT integration were not mainly about how to use technology but about how to apply ICT into pedagogies, classroom interaction and monitoring. This discrepancy between TK and other constructs could be attributed to the fact that TK is the primary form of knowledge while TCK, TPK, and TPACK are higher-level constitutes synthesised from the interaction between TK, PK, and CK (Mishra & Koehler, 2006). Developing TCK, TPK, and TPACK involves not only fluency and cognitive flexibility in technology, content, and pedagogy, but also contextual, pragmatic and nuanced understanding of their interrelation (Mishra & Koehler, 2006), needing more time and effort for EFL teachers to develop.

Furthermore, the EFL teachers' imbalanced development of TPACK components could also result from the professional development programs for COVID-19 ERT. The formal training from schools, which most of the participants (74.7%) received, generally aimed at all teachers, who taught in different subjects. Therefore, to quickly build COVID-19 ERT, the professional training for ICT in many schools mainly focused on how to use software and online platforms (Zhou et al., 2020), lacking specific training in EFL teaching or teaching pedagogy. Consequently, teachers might acquire more knowledge on using ICT from training but less on *applying* it in their online teaching. The comments of the open-ended question support this explanation. Many EFL teachers voiced their demands for supports and further school training to solve pedagogical problems in technological usage so that ICT integration could be “more than a mere formality” required by the government, as a participant stated in their response. Somehow, teachers regarded this COVID-19 crisis as a chance to make a change, therefore, being eager for the innovation and improvement of teaching that they believed ICT could bring. However, they were finally disappointed at their incapability of exploiting the full potentials of ICT owing to the limitation as an individual and insufficient direct assistance from institutions and governments. For these reasons, there should be a more problem-solving type of training that can simulate actual teaching contexts and include teachers' needs for TCK, TPK, and TPACK in the future.

A note of caution is due here for the combined factor TPK-TPACK. It indicated that the EFL teachers were unable to distinguish the items in these two groups. Although it is common in TPACK surveys that TPK and TPACK merge (see, e.g., Koh et al., 2010; Q. Liu et al., 2015), this could be partly because TPK and TPACK items in this study were not developed specifically for Chinese EFL teachers. Therefore, in future studies, a TPACK instrument for Chinese EFL teachers needs to be designed, and cautions need to be taken to ensure the reliability and validity of such an instrument.

### RQ3: Correlations Between ICT Acceptance and ICT Literacy

**Figure 4.**
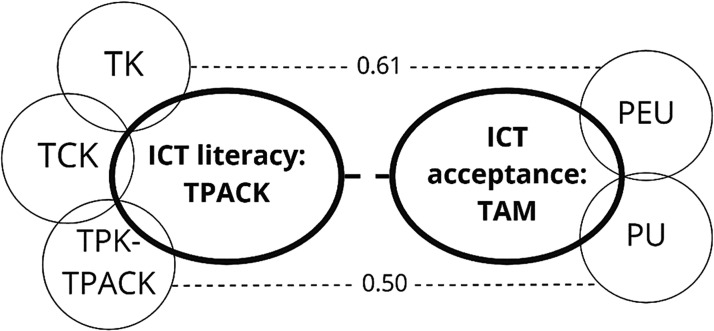
Relationships Between TAM and TPACK.

The third research question aimed to explain the underlying mechanism of EFL teachers' interaction with ICT. By examining the interaction between TPACK and ICT acceptance with correlation analysis, it was found that teachers' acceptance and TPACK perceptions are generally closely related, see Figure 4. That is to say, EFL teachers who have a higher level of TPACK may find it easier to use ICT and perceive ICT as a more useful tool. These results align with previous studies of significant correlations between TPACK and TAM (Hsu, 2016; Yang et al., 2019). They imply that professional development in developing TPACK could be provided to overcome the reluctance to learn ICT.

Two pairs of variables are found to be strongly correlated. The first correlation is between TK and PEU, indicating that teachers' knowledge of using ICT may be related to how easy they find using ICT. Secondly, TPK-TPACK is found to be strongly associated with PU. That is to say, the ability to integrate ICT into pedagogy and content may relate to perceptions of how useful ICT is. Given that the researchers of previous studies agreed that TPACK appears to influence ICT acceptance (see, e.g., Hsu, 2016; Yang et al., 2019), it may be inferred that a lower level of TPK-TPACK may in part result in a lower PU, while higher TK may contribute to a greater affirmation of PEU.

The implication of the correlation between PU and TPK-TPACK could partly explain the discrepancy of PU between previous literature and this study, as mentioned earlier in this section. In that part, I found that the participants tended to show lower PU than PEU in this research. To explain the reason, I assume that teachers' lower PU scores might be related to the inconsistency of the pedagogies between face-to-face teaching and online teaching. Teachers, therefore, need to develop innovative online teaching pedagogy - that is, TPK. When teachers failed to use their classroom teaching strategies in the new online teaching, or, we could say when they did not have enough TPK for teaching online courses, they might perceive this failure of transition as deficits of ICT, thus have less PU. The positive correlation of TPK and PU could support this explanation.

Previous research into the interaction between TPACK and TAM only limits to one component of TPACK to PU and PEU (Yang et al., 2019). The results of the current study expand the understanding of this interaction by showing that more components of TPACK are correlated to PU and PEU. However, it is important to bear in mind that correlation analysis cannot calculate causal relationships. Therefore, more research is needed for clarifying the causality.

## Conclusion

Ongoing ERT during the COVID-19 pandemic has challenged teachers in many ways, necessitating teachers' higher level of ICT integration to cope with the crisis. This investigation aimed to understand whether high school EFL teachers were ready for ICT during COVID-19 in mainland China. The study has found that the high school EFL teachers had an overall positive acceptance of ICT and perceptions of TPACK. Taken together, these results suggest that teachers might be quite ready for the ICT integration during COVID-19 ERT, considering the unforeseen challenges that are attendant with such an unprecedented scenario.

Especially, ERT requires teachers to develop technological knowledge for the wide adoption of ICT tools. The results show that the high school EFL teachers in China generally perceived that it was easy to use ICT and expressed their confidence in using technological tools, which implies the general effectiveness of COVID-19 ICT professional development. However, teachers found more obstacles in responding to the challenges of innovative teaching. The participants were not confident in integrating ICT in pedagogy and subject matter, which tend to lead to their concerns that the usefulness of ICT might mostly be limited to blended courses rather than *fully* online courses.

Also, the investigation shows that EFL teachers' TPACK perceptions and acceptance of ICT are closely correlated. Especially, how EFL teachers use ICT is strongly related to how they perceive the easiness of using ICT; and to what extent teachers could integrate ICT in their pedagogy and content teaching is associated with their perceptions of ICT usefulness.

### Limitations

A limitation of this study is that only the core factors from TAM and TPACK were included for investigation in this research. Further study could be carried out to ascertain possible effects and correlations for other components (e.g., PK, CK, PCK). Second, although the snowball sampling recruitment method was most appropriate for this research, a potential bias was introduced since interpersonal network might not present a good representation of the population of teachers who had taught online in COVID-19 ERT, therefore limited the data analysis. In addition, the sample had more participants aged under 40, so the profile of this sample may not be representative enough in the age group, which may reduce confidence in the generalisation of the findings.

### Implications and Future Studies

The findings of this study have several implications for teachers' ICT professional development. First, the results of this study reveal Chinese EFL teachers' rather positive adaption to an unfamiliar mode of online teaching – ERT and their resilience in the presence of the COVID-19 crisis. This presumable success in ICT integration could inform policymakers and teacher educators about the feasible strategies and training for the quick launch of ICT-based educational emergency response and contingency plans for a future crisis. Given the ICT learning approaches reported by the participants, it seems that short-term ICT training workshops, online resources and collaboration among colleagues could be the major pathways to guide teachers during the transition.

In addition, this study exposes the imbalance of Chinese EFL teachers' ICT professional development in a way that they might have gained skills in using technology but still need further advancement in adopting ICT in pedagogical and subject instruction, which could be prominent for teachers to provide quality ICT-based teaching. This highlights that the one-size-fits-all approach of ERT ICT training (i.e., training of TK) may be insufficient. Diverse contexts of teaching could be the focus of future targeted professional training.

It should be noted that teachers show awareness of the differences between blended courses and online courses, while the latter is believed to be less effective considering the professional development should also provide EFL teachers knowledge of the differences between blended courses and online courses, and help them develop newer roles and strategies to face such challenges as computer-mediated communication and online classroom management. These efforts could help mitigate the misconception of online education as an ineffective teaching mode and facilitate teachers' future technology integration.

Theoretically, this study extends the interrelation between TAM and TPACK by showing the two strong correlations (between PU and TPK-TPACK, and between PEU and TK). This could be meaningful to teacher training, implying that the co-development of literacy and ICT acceptance may be more efficient. It also accentuates the importance to consider the cognitive and psychological factors of ICT integration in tandem for future research.

Some further extension to this study could be to compare the readiness of ERT between different demographic backgrounds, such as countries, to explore how cultural and contextual factors influence teachers' integration of ICT in emergencies. Also, longitudinal studies can be carried out to investigate if these perceptions of ICT during ERT translate to face-to-face teaching when education provision is back to normalcy. Third, the correlation between TPACK and TAM could be further explored by more complex analysis such as structural equation modelling or qualitative studies.
